# COMMUNITY OUTCOMES FROM A PSYCHOSOCIAL INTERVENTION FOR LONELINESS AND SOCIAL ISOLATION IN OLDER ADULTS

**DOI:** 10.1093/geroni/igac059.2622

**Published:** 2022-12-20

**Authors:** Matthew Amick, Olivia Gartlan, Max Zubatsky, Marla Berg-Weger

**Affiliations:** Saint Louis University, St. Louis, Missouri, United States; Saint Louis University, St. Louis, Missouri, United States; Saint Louis University, St. Louis, Missouri, United States; Saint Louis University, St. Louis, Missouri, United States

## Abstract

Social isolation and loneliness are quickly becoming global health epidemics, where older adults are experiencing greater chronic health conditions and other complications as a result of this trend. This prevalence is likely underreported, given that stigma and health disparities associated with social isolation and loneliness often prevent older adults from reporting this to family members and professionals. This study's aim is to determine the rates of older adult social isolation and social connections from a psychosocial group model called Circle of Friends (CoF). Circle of Friends is built on a model of group rehabilitation model, with the aim to alleviate and prevent loneliness in older adults. The group protocol consists of 6-8 older adults who self-identify as lonely or socially isolated, who meet 12 times over three months. A midwestern university conducted virtual CoF groups between 2020-2022 to underserved areas of a metropolitan city. Researchers used the UCLA Loneliness Scale and Lubben Social Support Scale to determine differences in rates of loneliness and social supports. 15 members participated in three virtual CoF groups, with over 50% of the sample reporting low socioeconomic living conditions. Preliminary findings show that group members reported a 3-point decrease in overall loneliness (p<.05), but no differences in frequency of social supports through friends or family. While our research group continues to collect more data on outcomes of our CoF groups, these initial results highlight the need for more community interventions and social connection resources for older adults lacking financial means for healthcare options.

